# Jinbei Decoction Attenuates LPS‐Induced Acute Lung Injury via Suppression of TRAF6‐Dependent Inflammatory Response in Macrophage

**DOI:** 10.1111/jcmm.70944

**Published:** 2025-11-13

**Authors:** Wei Li, Aijun Zhang, Yongqing Cai, Haoyu Sun, Yao Teng, Zhaoqing Meng, Weiwei Zhou, Ruixin Liu, Zhen Zhang, Jingzhen Tian, Xia Li

**Affiliations:** ^1^ Innovative Institute of Chinese Medicine and Pharmacy Shandong University of Traditional Chinese Medicine Jinan China; ^2^ Key Laboratory of Traditional Chinese Medicine Classical Theory, Key Laboratory of Traditional Chinese Medicine Immunoregulation of Jinan, Traditional Chinese Medicine Immunoregulation Engineering Research Center of Shandong Province, Ministry of Education Shandong University of Traditional Chinese Medicine Jinan China; ^3^ Shandong Hongji‐Tang Pharmaceutical Group Co., Ltd. Jinan China; ^4^ School of Pharmacy Henan University of Chinese Medicine Zhengzhou China; ^5^ College of Traditional Chinese Medicine Shandong University of Traditional Chinese Medicine Jinan China

**Keywords:** acute lung injury, astrapterocarpan, Jinbei decoction, LPS, macrophage

## Abstract

Acute lung injury (ALI) is a life‐threatening inflammatory disease of the respiratory system, characterised by high mortality rates and lack of effective clinical interventions. Emerging evidence suggests that traditional Chinese medicine (TCM) formulations may offer therapeutic benefits in managing inflammatory respiratory diseases. Jinbei decoction (JBD), a 12‐herb TCM preparation currently used for pulmonary fibrosis, has shown preliminary therapeutic potential in ALI; however, mechanistic studies remain limited. This study systematically evaluated JBD's therapeutic efficacy and elucidated its molecular mechanisms in LPS‐induced ALI. Survival analysis demonstrated that JBD significantly improved survival rates in a concentration‐dependent manner, while histopathological evaluation revealed a marked reduction in pulmonary tissue damage. These effects were further supported by significant decreases in circulating levels of major pro‐inflammatory cytokines, such as TNF‐α, IL‐6 and IL‐1β. Network pharmacology analysis identified 111 molecular targets associated with ALI pathogenesis influenced by JBD components, highlighting the regulatory effect on inflammatory signalling pathways in macrophages as the key intervening mechanism. Specifically, JBD suppressed LPS‐induced inflammatory responses by inhibiting ERK phosphorylation and blocking IKKα/β activation, thereby preventing NF‐κB‐dependent cytokine production in macrophages. Notably, astrapterocarpan was identified as the primary bioactive constituent of JBD through integrated network pharmacology and biochemical analyses. It was found to directly destabilise TRAF6 protein and to exhibit therapeutic efficacy comparable to that of dexamethasone in promoting histological recovery. In vivo experiments further confirmed that JBD significantly reduced TRAF6 expression in murine models, reinforcing the conclusion that its therapeutic effects are predominantly mediated by astrapterocarpan. Collectively, these findings suggest that JBD functions as an agent capable of regulating macrophage polarisation and mitigating cytokine storm through TRAF6‐dependent signalling pathways, thereby providing a mechanistic basis for its potential clinical application in inflammatory lung diseases.

## 1 Introduction

Acute lung injury (ALI) is a severe pulmonary disorder defined by the sudden onset of profound hypoxemia and impaired respiratory function. Epidemiological evidence indicates that ALI impacts roughly three million people globally every year, and although there have been considerable improvements in treatment methods, it still carries a mortality rate of nearly 43% [1, 2]. According to the Global Burden of Disease Study, ALI contributes to nearly 20% of global respiratory‐related deaths, highlighting its substantial burden on healthcare infrastructure [3]. Sepsis is the most common underlying cause, accounting for over 80% of clinical cases [4, 5]. A key pathogenic factor in sepsis‐related ALI is lipopolysaccharide (LPS), an essential constituent of gram‐negative bacterial cell walls that triggers robust inflammatory reactions and compromises the structural and functional integrity of pulmonary endothelial and epithelial cells. This disruption facilitates leukocytes, especially macrophages and neutrophils, to migrate into the pulmonary interstitium and amplifies LPS or injury signal‐induced inflammatory responses within lung parenchyma [6, 7].

Inflammatory responses in the vasculature are linked to systemic immune dysregulation and drive ALI progression through sustained cytokine production [8, 9]. Macrophages, key players in innate immunity, normally balance immune surveillance, host defence and tissue repair, but these functions are impaired in ALI [10, 11]. Prolonged exposure to LPS, and further damage‐associated molecular patterns (DAMPs) shifts macrophages from a reparative to a pro‐inflammatory state, characterised by excessive secretion of TNF‐α, IL‐6 and IL‐1β, leading to persistent inflammation and tissue injury [12, 13, 14]. Key to this process is TLR4/TRAF6/NF‐κB pathway activation [15]. When TLR4 detects LPS or danger signals, it triggers a coordinated sequence of intracellular events. These signals are mainly transmitted via the MyD88‐dependent pathway, which relays inflammatory signals from the receptor to the nucleus [16, 17]. This pathway involves IRAK recruitment, TRAF6 ubiquitination and formation of the TAK1/TAB complex. These events activate two major downstream pathways: IKKβ‐mediated NF‐κB activation and MAP3K‐dependent JNK/p38/ERK signalling [18, 19, 20]. Sustained NF‐κB activity drives neutrophil infiltration, epithelial cell death, and extracellular matrix breakdown, leading to irreversible alveolar damage [21, 22].

At present, all established therapeutic strategies for ALI remain predominantly supportive, with lung‐protective ventilation serving as a cornerstone of clinical management [23]. This ventilatory strategy facilitates alveolar recruitment while mitigating the risk of pulmonary overdistension through coordinated application of positive end‐expiratory pressure (PEEP) and reduced tidal volumes [24]. However, the spatial heterogeneity of pulmonary lesions poses a significant challenge in determining optimal PEEP levels that can effectively prevent regional alveolar collapse, despite its widespread clinical application. Although supportive interventions may improve initial oxygenation by promoting pulmonary blood flow redistribution and alveolar recruitment [25], their therapeutic efficacy is largely confined to advanced fibrotic stages, where structural alterations have become irreversible. Corticosteroids, such as dexamethasone are considered first‐line anti‐inflammatory agents due to their capacity to inhibit NF‐κB‐mediated proinflammatory cytokine production [26, 27]; however, their clinical utility is limited by substantial interindividual variability in pharmacokinetic and pharmacodynamic profiles. Thus, current therapeutic approaches for ALI remain constrained by inconsistent clinical outcomes and notable adverse effects associated with interventions [28]. Given these persistent limitations, the development of novel therapeutic approaches that integrate multi‐target efficacy with reduced systemic toxicity has emerged as a critical priority in ALI research and clinical practice.

Jinbei decoction (JBD), a traditional Chinese medicine (TCM) preparation containing twelve herbal components, including Astragali Radix, Codonopsis Radix, Glehniae Radix, Salviae Miltiorrhizae Radix et Rhizoma, Angelicae Sinensis Radix, Chuanxiong Rhizoma, Lonicerae Japonicae Flos, Forsythiae Fructus, Scutellariae Radix, Fritillariae Cirrhosae Bulbus, Pinelliae Rhizoma Praeparatum Cum Alumine and Glycyrrhizae Radix et Rhizoma, is an empirically derived formula that has been clinically applied in the management of interstitial lung disease. Emerging evidence indicates that JBD exhibits therapeutic potential in modulating oxidative stress and inflammatory pathways associated with idiopathic pulmonary fibrosis (IPF). Mechanistic studies have demonstrated that JBD exerts anti‐fibrotic effects through a network of interrelated processes, including the enhancement of synergistic anti‐inflammatory activity, the inhibition of extracellular matrix accumulation and the restoration of immune homeostasis via regulation of Th17/Treg balance [29, 30, 31]. Notably, preclinical data imply that JBD significantly attenuates pathological tissue remodelling while maintaining pulmonary functional integrity. However, its therapeutic application in ALI remains to be elucidated.

To assess the protective effects of JBD in mitigating acute inflammatory responses induced by LPS, we performed comprehensive experimental investigations focusing on survival outcomes, cytokine profile modifications and histopathological alterations in lung tissue. Through mechanistic analysis, astrapterocarpan was identified as the key bioactive constituent responsible for JBD's anti‐inflammatory properties, exhibiting a regulatory function in the TLR4/NF‐κB signalling pathway in macrophages. These findings provide novel evidence supporting the therapeutic efficacy of JBD in managing ALI triggered by LPS and suggest astrapterocarpan as a promising agent for future clinical exploration.

## 2 Methods

### 2.1 Chemicals and Reagents

The compounds baicalin (HY‐N0197), wogonin (HY‐N0400), chlorogenic acid (HY‐N0055), glycyrrhizin (HY‐N0184), phenylalanine (HY‐N0215) and astrapterocarpan (HY‐N2484) were sourced from MedChemExpress. Cell culture reagents, such as RPMI 1640 medium (C3001‐0500), DMEM (C3103‐0500), FBS (C04001‐500) and penicillin/streptomycin solution (C3420‐0100) were procured from VivaCell (Shanghai, China). Protein concentration measurements were conducted using the BCA assay kit (P0012) provided by Beyotime (Shanghai, China). ELISA kits were purchased from Multi Sciences (Hangzhou, China) for IL‐6 (EK206), TNF‐α (EK282) and IL‐1β (EK201B). LPS (L8880) was acquired from Solarbio (Beijing, China). Immunoblotting antibodies targeting GAPDH (ab8245) and phospho‐IKK‐alpha/beta (ab194528) were obtained from Abcam (Cambridge, MA, USA). Phosphorylated ERK (4370), total ERK (4695), phosphorylated P65 (3033) and antibody against P65 (8242) were sourced from Cell Signalling Technology.

### 2.2 Preparation of JBD

The JBD is formulated according to the following standardised procedure: Astragali Radix, Codonopsis Radix, Glehniae Radix, Salviae Miltiorrhizae Radix et Rhizoma, Angelicae Sinensis Radix, Chuanxiong Rhizoma, Lonicerae Japonicae Flos, Forsythiae Fructus, Scutellariae Radix, Fritillariae Cirrhosae Bulbus, Pinelliae Rhizoma Praeparatum Cum Alumine and Glycyrrhizae Radix et Rhizoma are precisely weighed in equal proportions (1:1:1:1:1:1:1:1:1:1:1:1). The manufacturing process begins with a preliminary treatment stage, in which the volatile compounds are obtained from Angelicae Sinensis Radix, Chuanxiong Rhizoma and Forsythiae Fructus via steam distillation. Meanwhile, the remaining herbal materials are subjected to two rounds of water decoction. The obtained decoction undergoes concentration under reduced pressure, and is subsequently purified through a series of steps involving alcohol precipitation and water precipitation to eliminate impurities. The volatile extract collected from distillation is then merged with the purified aqueous solution. Finally, the liquid is passed through a 0.22 μm membrane filtration system to yield a filtrate suitable for oral administration, with an herbal concentration of 0.712 g/mL based on the original raw herbal materials used in the formulation.

### 2.3 Preparation of Serum Containing Drug for JBD

Following 1 week of acclimatisation, male Sprague–Dawley (SD) rats were randomly allocated into three separate treatment groups. The intragastric doses of JBD for rats were calculated based on equivalent dose conversion from the clinical dose in humans (60 mL/60 kg daily). The three groups received different dosage regimens of JBD via intragastric administration: a low‐dose group (3.15 mL/kg), a medium‐dose group (6.3 mL/kg) and a high‐dose group (12.6 mL/kg). The control group was given an equal volume of normal saline following the same administration procedure. On the seventh day, immediately after the final dosing, blood samples were collected via puncture of the abdominal aorta. The collected blood samples were subsequently centrifuged to separate the supernatant. The resulting serum was then heat‐inactivated, filtered and maintained at −20°C for future analytical evaluation.

### 2.4 Mice and LPS Induced ALI Model

Male C57BL/6J mice and SD rats (8 weeks of age; obtained from HFK Bioscience, Beijing) were acclimatised to the laboratory environment for 72 h prior to the initiation of the experiments. The animals were maintained under controlled conditions in a pathogen‐free facility. All experimental work adhered to the ethical principles and procedural standards outlined by the Institutional Animal Care and Use Committee (IACUC) of Shandong University of Traditional Chinese Medicine. The animals were kept in sterile polypropylene cages with regulated environmental parameters: a temperature of 22°C ± 2°C, humidity of 50% ± 5%, and a 12‐h light–dark rhythm. After the acclimation period, the mice were randomly divided into five separate groups: (1) Control (receiving PBS), (2) LPS treated, (3) LPS combined with low‐dose JBD (JBD‐Low, 6.15 mL/kg), (4) LPS combined with medium‐dose JBD (JBD‐Medium, 12.3 mL/kg), and (5) LPS combined with high‐dose JBD (JBD‐High, 24.6 mL/kg). To generate a model of systemic inflammation, animals in groups 2–5 received intraperitoneal injections of LPS at a dosage of 5 mg per kilogram of body weight. Groups 3–5 received daily oral gavage of JBD at low, medium, or high concentrations, respectively, for 7 days before LPS administration. Blood samples were collected 6 h after LPS injection by puncturing the retro‐orbital sinus under anaesthesia, using sterile techniques. After centrifugation, the upper serum layer was carefully retrieved and preserved at −20°C for subsequent biochemical testing. Immediately after blood collection, all animals were euthanized humanely, and lung tissues were removed in a systematic manner for histopathological and molecular analysis. In a separate set of experiments evaluating astrapterocarpan, inflammation was initiated via intraperitoneal injection of LPS at a dosage of 5 mg/kg, with PBS‐treated animals used as controls. Astrapterocarpan was given at doses of 5 or 10 mg/kg via intraperitoneal injection 2 h after LPS administration.

### 2.5 Isolation of Primary Murine Alveolar Macrophages

Alveolar macrophages were extracted from the pulmonary tissue of C57BL/6 mice using bronchoalveolar lavage (BAL) following the execution of pre‐established experimental procedures. Animal euthanasia was carried out following institutional ethical standards for laboratory animal handling. The trachea was surgically uncovered and cannulated with an 18‐gauge needle to facilitate lavage. Each lung was irrigated repeatedly with 1 mL of chilled PBS supplemented with 0.5 mM EDTA, with a 60‐s pause before each aspiration to enhance fluid retrieval. The collected lavage fluid was subjected to centrifugation at 300 × g for 10 min at a temperature of 4°C. The resulting cell pellet was resuspended in RPMI‐1640 culture medium supplemented with 10% foetal bovine serum (FBS) and 1% penicillin–streptomycin. The cellular suspension was subsequently transferred into 6‐well culture plates and maintained under controlled incubation conditions (37°C, 5% CO_2_) for 2 h to enable macrophage attachment. Unattached cells were eliminated through two mild PBS washes, leaving only the firmly adhered alveolar macrophages for downstream experimental assessment.

### 2.6 Cell Culture and Treatment

RAW264.7 cells were maintained in Dulbecco's Modified Eagle Medium (DMEM) supplemented with 10% heat‐inactivated FBS and antibiotics, including 100 U/mL penicillin and 100 μg/mL streptomycin. To isolate primary peritoneal macrophages (PEMs), C57BL/6 mice were given an intraperitoneal injection of 4% thioglycollate broth (Sigma‐Aldrich, T9032). Peritoneal macrophages were harvested 72 h post‐injection through lavage and subsequently maintained in RPMI 1640 medium. All cell cultures were maintained at 37°C in a humidified incubator containing 5% CO_2_.

To investigate the anti‐inflammatory properties of JBD, cells were incubated for 24 h with either control medium or that supplemented with low, medium or high doses of JBD before being stimulated with LPS. After a 6‐h LPS exposure, culture supernatants were collected to quantify cytokine release using enzyme‐linked immunosorbent assay (ELISA). In parallel, total RNA was extracted for quantitative real‐time PCR (qRT‐PCR) to measure gene expression levels. To further explore how JBD modulates LPS‐induced signalling pathways, cells were pretreated with either PBS or high‐concentration JBD‐containing serum for 24 h, followed by stimulation with LPS (100 ng/mL) at multiple time points (0, 15, 30 and 60 min). Cell lysates were then prepared and analysed by immunoblotting to detect the phosphorylation status of essential signalling molecules.

### 2.7 Constituent Analysis of JBD by UHPLC–MS/MS

Liquid chromatography‐mass spectrometry (LC–MS) and tandem mass spectrometry (MS/MS) were carried out using a Waters ACQUITY UHPLC system (H‐Class, Milford, MA, USA) interfaced with a Bruker Impact II Q‐TOF mass spectrometer (Bremen, Germany). Chromatographic separation was conducted on an Agilent SB‐C18 column (2.1 × 100 mm, 1.8 μm; Santa Clara, CA, USA), employing a binary mobile phase consisting of (A) 0.1% (v/v) formic acid in water and (B) acetonitrile. The gradient elution profile was set as follows: 0–4 min, 5%–10% B; 4–20 min, 10%–30% B; 20–27 min, 30%–100% B; 27–30 min, maintained at 100% B; 30–30.1 min, decreased to 5% B; and 30.1–35 min, held at 5% B. The column temperature was kept at 40°C, with a flow rate of 0.3 mL/min and an injection volume of 5 μL.

Mass spectrometric analysis was carried out in both positive and negative electrospray ionisation (ESI) modes. The ESI source parameters were set to a capillary voltage of 3500 V in positive mode and 3000 V in negative mode. The Q‐TOF mass spectrometer was operated under optimised conditions, including a drying gas flow of 8 L/min, nebuliser pressure of 2.0 Bar, and desolvation gas temperature of 220°C. Additional instrumental settings included a collision RF amplitude of 750 Vpp, pre‐pulse storage time of 6 μs, and ion transfer time of 80 μs. Full‐scan mass data were acquired across an *m/z* range of 50–1500. Molecular formulas were determined using Bruker DataAnalysis 5.0 software, with a mass accuracy tolerance of < 10 ppm.

### 2.8 Network Pharmacology Analysis

Therapeutic targets associated with LPS‐induced ALI were identified through a comprehensive and systematic search of publicly accessible databases, including GeneCards, the Comparative Toxicogenomics Database (CTD) and the Gene Expression Omnibus (GEO). To predict the potential molecular targets of the bioactive compound JBD, a range of computational prediction tools, including HIT2.0, STITCH, SwissTargetPrediction, DrugBank and the Similarity Ensemble Approach (SEA), were rigorously applied. Pathway enrichment analysis was carried out using the KEGG database to identify key biological pathways potentially regulated by JBD in LPS‐induced ALI. Functional annotation and classification were performed utilising the DAVID bioinformatics platform (https://david.ncifcrf.gov/), adopting a statistical significance threshold of *p* < 0.05.

### 2.9 Major Ingredient Screening

The compound‐sepsis target network of JBD's bioactive components was systematically constructed and analysed using Cytoscape software. To evaluate the topological properties and identify key network hubs, the CytoNCA and CytoHubba plugins were employed. These computational tools facilitated an in‐depth analysis of the centrality and functional relevance of both the compounds and their associated targets within the context of sepsis regulation.

### 2.10 Enzyme‐Linked Immunosorbent Assay (ELISA)

Concentrations of inflammatory cytokines, including IL‐6, IL‐1β and TNF‐α, were measured in the cell culture supernatants using established enzyme‐linked immunosorbent assay (ELISA) protocols in accordance with the manufacturer's instructions. The analysis was carried out using commercial detection kits purchased from Multi Sciences (Hangzhou, China).

### 2.11 Haematoxylin–Eosin (H&E) Staining

Lung tissue specimens were first washed with cold PBS and subsequently fixed in a 4% paraformaldehyde solution. After fixation, the tissues were processed for paraffin embedding using standard techniques, and 4 μm sections were obtained with a microtome. The prepared tissue sections were then subjected to H&E staining to facilitate histopathological examination. Changes in the tissue architecture were assessed systematically and documented using digital microscopy with a light microscope (E100, Nikon, Japan).

### 2.12 Western Blot Analysis

Protein lysates were prepared using RIPA buffer supplemented with protease inhibitors (Solarbio), and the total protein concentration was determined using a bicinchoninic acid (BCA) protein assay kit (Beyotime). Equal amounts of protein (20 μg per lane) were loaded onto 10% sodium dodecyl sulfate‐polyacrylamide gel electrophoresis (SDS‐PAGE) gels and separated by electrophoresis. The resolved proteins were then transferred onto nitrocellulose membranes (Merck Millipore, USA). Following transfer, the membranes were incubated in a blocking solution containing 5% non‐fat dry milk in PBST buffer (0.1% Tween‐20) for 1 h at room temperature to reduce background binding. The membranes were then incubated with primary antibodies overnight at 4°C under gentle agitation. After three 10‐min washes with PBST, the membranes were treated with species‐matched horseradish peroxidase (HRP)‐conjugated secondary antibodies for 1 h at room temperature. Bound antibody complexes were detected using Pierce Enhanced Chemiluminescence (ECL) substrate and visualised with a chemiluminescence imaging system.

### 2.13 Quantitative Real‐Time PCR

Total RNA was extracted using TRIzol reagent (CWBIO, China) according to the manufacturer's instructions. Complementary DNA (cDNA) was synthesised from 1 μg of total RNA through reverse transcription. Quantitative real‐time PCR was performed using SYBR Green Master Mix (CWBIO) on a real‐time PCR system. The expression levels of the target genes were normalised to internal reference genes and calculated using the 2^−ΔΔ^Ct method. The primer sequences (5′ → 3′) used in this study are provided as follows:

*TNFA* ( *H. sapiens* ): forward, CCTCTCTCTAATCAGCCCTCTG; reverse, GAGGACCTGGGAGTAGATGAG;

*Tnfa* ( *M. musculus* ): forward, CAGGCGGTGCCTATGTCTC; reverse, CGATCACCCCGAAGTTCAGTAG;

*IL6* ( *H. sapiens* ): forward, ACTCACCTCTTCAGAACGAATTG; reverse, CCATCTTTGGAAGGTTCAGGTTG;

*Il6* ( *M. musculus* ): forward, CTGCAAGAGACTTCCATCCAG; reverse, AGTGGTATAGACAGGTCTGTTGG;

*IL1B* ( *H. sapiens* ): forward, TTCGACACATGGGATAACGAGG; reverse, TTTTTGCTGTGAGTCCCGGAG;

*Il1b* ( *M. musculus* ): forward, GAAATGCCACCTTTTGACAGTG; reverse, TGGATGCTCTCATCAGGACAG;

*GAPDH* ( *H. sapiens* ): forward, GGAGCGAGATCCCTCCAAAAT; reverse, GGCTGTTGTCATACTTCTCATGG;

*Gapdh* ( *M. musculus* ): forward, AGGTCGGTGTGAACGGATTTG; reverse, GGGGTCGTTGATGGCAACA.

### 2.14 Dual Luciferase Reporter Assay

Plasmids were introduced into RAW264.7 cells using Lipofectamine 3000 (Thermo Fisher). The plasmid constructs used included the NF‐κB luciferase reporter plasmid and the pRL‐TK Renilla luciferase control vector. These were co‐transfected along with plasmids expressing the proteins MyD88, IRAK4, TRAF6 and TAK1. Luciferase activity was measured using a dual luciferase assay kit (Promega, Madison, WI, USA) in conjunction with a Luminoskan Ascent luminometer (Thermo Scientific, Waltham, MA, USA). All luciferase assays were carried out in 24‐well plates.

### 2.15 Molecular Docking Analysis

The three‐dimensional structures of the main bioactive compounds in JBD were sourced from the PubChem database, whereas the related target protein structures were obtained from the protein data bank (PDB) repository. Receptor protein preparation began with the elimination of water molecules and irrelevant ligands using PyMOL 8 software (Schrodinger LLC; version 2.5.2). Additional refinement procedures, such as adding hydrogen atoms and normalising charges, were carried out using AutoDockTools (Scripps Research Institute; release 1.5.7), after which the refined biomolecules were transformed into pdbqt format to facilitate molecular docking simulations. Virtual screening based on structural data was carried out using the AutoDock Vina platform (Scripps Research Institute; v1.2.3), which systematically analysed ligand‐receptor interactions by evaluating spatial compatibility and binding energy parameters. The computational workflow examined both steric fit and thermodynamic stability during the formation of protein‐ligand complexes, and validation steps were implemented to ensure the reliability and repeatability of the docking protocol. Validation was carried out by redocking the original ligand into its designated binding site on the protein. The binding free energy (ΔG, kcal/mol) was calculated for the most stable molecular conformations, followed by visualisation and structural analysis of the resulting protein‐ligand complexes using PyMOL software.

### 2.16 CETSA Assay

The Cellular Thermal Shift Assay (CETSA) was applied to evaluate how compound binding affects the thermal stability of target proteins. In short, primary peritoneal macrophages (PEMs) or RAW264.7 cells were exposed to astrapterocarpan (10 μM) or DMSO control for a duration of 24 h. Following treatment, cells were collected, washed with PBS and resuspended in ice‐cold PBS containing a protease inhibitor cocktail. Complete cell lysis was accomplished through three repeated cycles of freezing in liquid nitrogen and thawing at room temperature. Portions of the lysates were then subjected to a controlled heating process in a thermal cycler with gradual temperature elevation (37°C, 42°C, 47°C, 52°C and 57°C), maintaining each temperature for 3 min. After thermal treatment, cellular remnants were separated by centrifugation at 20,000 × g for 15 min at 4°C, and the clarified lysates were used for downstream processing. Protein concentration was determined using the BCA protein assay kit. Samples with equal amounts of protein were separated by SDS‐PAGE and then transferred onto nitrocellulose membranes for Western blot analysis.

### 2.17 Statistical Analysis

All experimental results were evaluated using GraphPad Prism 10.0 statistical software (GraphPad Inc., USA). Numerical data were reported as mean ± standard error of the mean (SEM). For comparing two unrelated groups, the unpaired Student's *t*‐test was applied, while one‐way analysis of variance (ANOVA) was used to assess variations across multiple groups. The Pearson correlation coefficient was calculated to investigate the linear dependence between continuous variables. Statistical significance for all inferential testing was set at a *p*‐value below 0.05.

## 3 Results

### 3.1 JBD Alleviates LPS‐Induced Acute Lung Injury by Inhibiting Inflammatory Cytokine Production and Potentially Regulating Macrophage Activity

**FIGURE 1:**
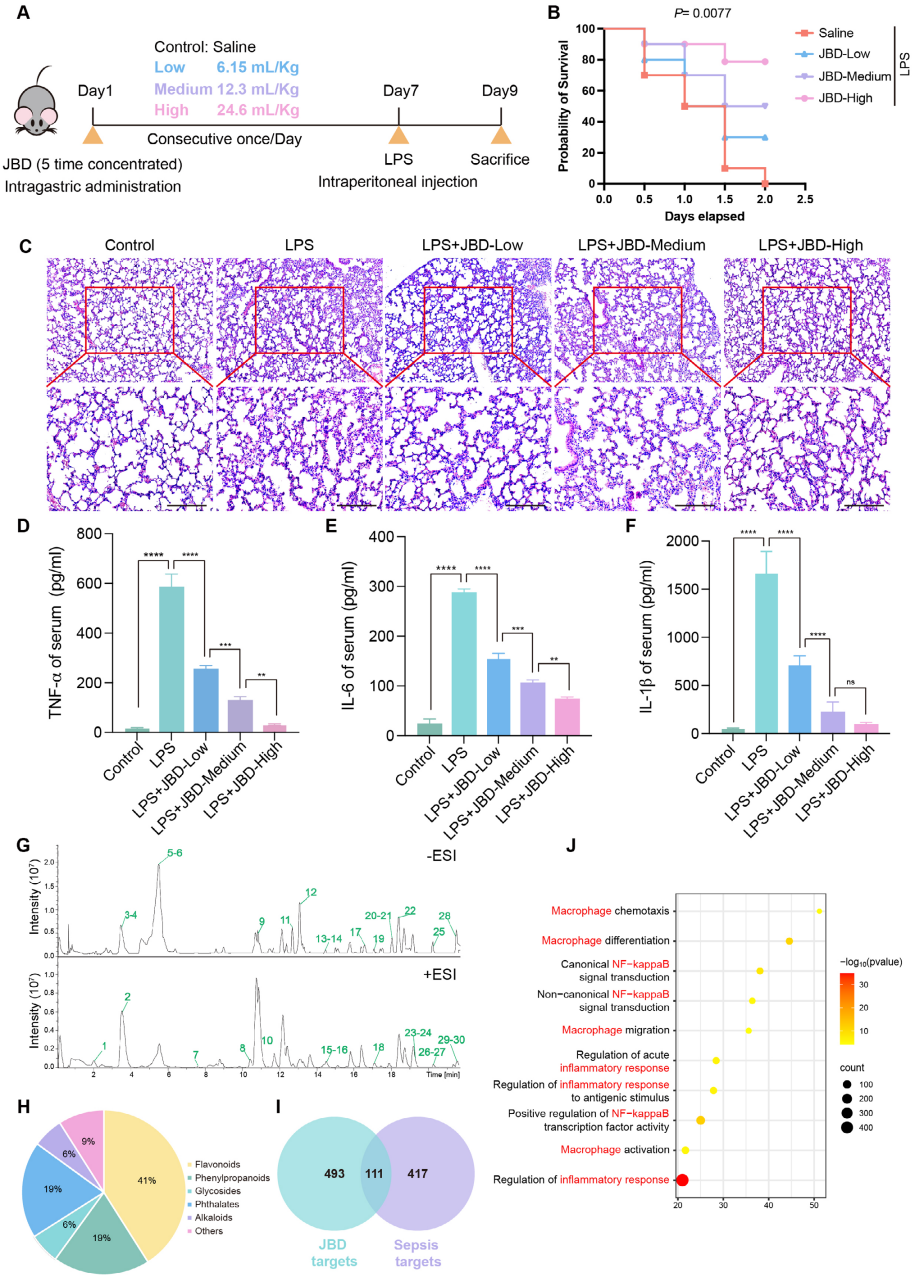
JBD alleviates LPS‐induced acute lung injury through anti‐inflammatory effects and macrophage regulation. (A) Diagram illustrating the experimental protocol for JBD pretreatment (varying dosage levels) followed by LPS challenge (5 mg/kg intraperitoneal administration) in C57BL/6 murine subjects (10 animals per cohort). (B) Survival probability analysis demonstrating enhanced viability correlated with JBD dosage gradients (Log‐rank comparative analysis, *p* = 0.0077 relative to LPS control). (C) Histopathological evaluation of lung tissue sections stained with haematoxylin–eosin (magnification scale: 100 μm) showing mitigated pathological manifestations including diminished alveolar septal hyperplasia, reduced leukocyte infiltration and attenuated hemorrhagic presentation following JBD administration. (D–F) Cytokine profile quantification (TNF‐α, IL‐6 and IL‐1β) in peripheral blood 6 h post‐LPS exposure, demonstrating concentration‐dependent anti‐inflammatory efficacy of JBD intervention. (G) Chromatographic analysis using UHPLC–MS/MS in dual ionisation modes (positive/negative) successfully characterised 30 bioactive constituents absorbed from JBD. (H) The phytochemical distribution revealed flavonoid derivatives as predominant (41%), followed by phenylpropanoid compounds (19%) through compositional analysis. (I) Target intersection analysis identified 111 shared biomarkers between JBD's 604 molecular targets and 528 acute lung injury‐associated genes. (J) Enriched KEGG pathways (−log_10_[*p*‐value] ranked) featured immune‐inflammatory regulators, including NF‐κB cascade, Toll‐like receptor signalling and cytokine networks. (Data presented as mean ± standard error; **p < 0.01, ***p < 0.001, ****p < 0.0001).

To investigate the therapeutic potential of JBD in mitigating ALI induced by LPS, a murine model of inflammatory pulmonary injury was established. Mice were pretreated with JBD via intragastric administration at low, medium and high doses for seven consecutive days prior to LPS challenge (Figure 1A). The results indicated that JBD pretreatment significantly improved survival rates in LPS‐exposed mice in a dose‐dependent manner (Figure 1B). Notably, mice receiving the highest dose of JBD exhibited an 80% increase in survival compared to the LPS‐only group, while the low‐dose group showed a 30% improvement. These results demonstrate that JBD offers substantial protection against LPS‐induced mortality in mice.

Building upon these findings, we conducted a histopathological analysis to further assess the protective role of JBD in LPS‐mediated ALI. Lung tissue sections were processed for H&E staining to assess structural alterations. Microscopic examination revealed a significant decrease in pathological damage in JBD‐pretreated mice, characterised by reduced alveolar septal thickening, diminished leukocyte infiltration and decreased vascular leakage (Figure 1C). Given that this murine model recapitulates the pathophysiological progression of acute inflammation‐driven lung injury leading to mortality, we proceeded to assess whether JBD modulates systemic inflammatory responses. The concentrations of pro‐inflammatory cytokines, including TNF‐α, IL‐1β and IL‐6, in serum were measured via ELISA. The data demonstrated that JBD administration significantly and dose‐dependently suppressed the production of these inflammatory mediators (Figure 1D–F). Collectively, these findings suggest that JBD attenuates LPS‐induced ALI, at least partially, by inhibiting systemic inflammatory activation.

We then adopted an integrated approach combining UHPLC–MS/MS and network pharmacology analysis to identify the absorbed constituents, potential targets and key signalling pathways involved in JBD's anti‐inflammatory effects, thereby further elucidating the biological mechanisms underlying JBD's protective role against LPS‐induced ALI. UHPLC–MS/MS analysis of JBD‐containing serum confirmed the presence of thirty absorbed compounds in the systemic circulation (Table 1, Figure 1G). Chemical classification indicated that flavonoids were the most abundant class (41%), followed by phenylpropanoids (19%), phthalates (19%), alkaloids (6%) and glycosides (6%) (Figure 1H), suggesting that these compounds may contribute to JBD's pharmacological activity. Using multiple databases (DrugBank, HIT2.0, SEA, STITCH and SwissTargetPrediction), we identified 604 potential protein targets of the absorbed JBD components. Concurrently, GeneCards, CTD and OMIM were employed to collect 528 known ALI‐ and sepsis‐related targets. Venn diagram analysis revealed 111 overlapping targets (Figure 1I), and KEGG pathway enrichment analysis was conducted, which demonstrated significant enrichment in key biological pathways, including inflammatory response, macrophage activation and NF‐κB signalling (Figure 1J). Taken together, JBD may regulate the inflammatory signalling pathways in macrophages, thereby exerting a protective effect against LPS‐induced ALI.

**TABLE 1:** Analysis of chemical components of drug‐containing plasma for Jinbei oral liquid.

NO.	Compound	Detection pattern	Formula	TR/min	Extraction mass (Da)	Found mass (Da)	MS/MS	Classify
1	Protocatechualdehyde	[M−H]^−^	C_7_H_6_O_3_	4.4000	137.0242	137.0243	119.0123; 108.0219; 81.0336	F
2	Malonic acid	[M−H]^−^	C_10_H_10_O_5_	4.5000	209.0450	209.0452	165.0557; 121.0655	F
3	Chlorogenic acid	[M−H]^−^	C_16_H_18_O_9_	5.8000	353.0872	353.0880	191.0556; 173.0444; 135.0441	B
4	1‐Caffeoylquinic acid	[M−H]^−^	C_16_H_18_O_9_	5.5000	353.0871	353.0878	191.0563; 161.0255	B
5	Liquiritin	[M−H]^−^	C_21_H_22_O_9_	10.9000	417.1185	417.1193	255.0666; 135.0082	A
6	Luteolin 7‐O‐glucoside	[M−H]^−^	C_21_H_20_O_11_	13.2000	447.0927	447.0932	151.0030; 107.0241	A
7	4,5‐Dicaffeoylquinic acid	[M−H]^−^	C_25_H_24_O_12_	12.6000	515.1185	515.1198	191.0615; 179.0350; 173.0436	B
8	Rosmarinic acid	[M−H]^−^	C_18_H_16_O_8_	14.2000	359.0765	359.0766	197.0442; 179.0345; 161.0243	B
9	3,4‐Dicaffeoylquinic acid	[M−H]^−^	C_25_H_24_O_12_	14.2000	515.1184	515.1196	353.0876; 191.0580; 173.0450	B
10	Baicalin	[M−H]^−^	C_21_H_18_O_11_	17.2000	445.0763	445.0777	269.0458; 223.0425; 169.0713; 113.0244	A
11	Liquiritigenin	[M−H]^−^	C_15_H_12_O_4_	16.5000	255.0660	255.0602	135.0083; 119.0506	A
12	Apigenin 7‐glucuronide	[M−H]^−^	C_21_H_18_O_11_	18.0000	445.0768	445.0774	269.0449; 171.0489; 113.0237	A
13	Chrysin −7‐O‐beta‐D‐glucuronoside	[M−H]^−^	C_21_H_18_O_10_	18.2000	429.0819	429.0826	253.0493; 175.0243; 113.0242	A
14	Oroxindin	[M−H]^−^	C_22_H_20_O_11_	18.4000	459.0927	459.0931	283.0609; 268.0372; 175.0245	A
15	9,12,13‐Trihydroxy‐10‐octadecenoic acid	[M−H]^−^	C_18_H_34_O_5_	22.8000	329.2324	329.2335	293.2134; 229.1453; 211.1342; 171.1043; 239.1468	F
16	Wogonin	[M−H]^−^	C_16_H_12_O_5_	24.0000	283.0604	283.0612	268.0380; 239.0350; 163.0044	A
17	Phenylalanine	[M+H]^+^	C_9_H_11_NO_2_	2.0000	166.0862	166.0863	120.0807; 103.0543	E
18	Tryptophan	[M+H]^+^	C_11_H_12_N_2_O_2_	3.6000	205.0970	205.0971	188.0703; 170.0598; 159.0914	E
19	Sweroside	[M+H]^+^	C_16_H_22_O_9_	7.6000	359.1338	359.1333	197.0808; 179.0699; 151.0755; 127.0388	F
20	Hydroxymethyl coumarin	[M+H]^+^	C_10_H_8_O_3_	10.2000	177.0546	177.0550	135.0421; 117.0335; 89.0395	B
21	Quercitrin	[M+H]^+^	C_21_H_20_O_11_	11.8000	449.1074	449.1080	287.0570; 153.0188	A
22	Senkyunolide I	[M+H]^+^	C_12_H_16_O_4_	14.3000	247.0939	247.0939	247.0953; 165.0573	D
23	Senkyunolide F	[M+H]^+^	C_12_H_14_O_3_	14.3000	207.1015	207.1014	207.1013; 189.0904; 179.1064; 161.0965; 133.1017	D
24	Astrapterocarpan	[M+H]^+^	C_17_H_16_O_5_	17.2000	301.1069	301.1074	167.0704	A
25	Butylphthalide	[M+H]^+^	C_12_H_14_O_2_	22.7000	191.1067	191.1067	191.1068; 173.0965; 145.1014	D
26	Senkyunolide G	[M+H]^+^	C_12_H_16_O_3_	22.7000	209.1175	209.1173	231.0991; 209.1176; 191.1074	D
27	Licoricesaponin G2	[M+H]^+^	C_42_H_62_O_17_	22.9000	839.4047	939.4051	663.3757; 487.3412; 469.3307	C
28	Glycyrrhizin	[M+H]^+^	C_42_H_62_O_16_	23.3000	823.4103	823.4112	647.3795; 471.3475; 453.3362	C
29	SenkyunolideA	[M+H]^+^	C_12_H_16_O_2_	24.6000	193.1222	193.1224	147.1166; 137.0597; 91.0540	D
30	Ligustilide	[M+H]^+^	C_12_H_14_O_2_	25.5000	191.1064	191.1067	173.0965; 163.1112; 155.0862	D

*Note:* A: flavonoid compounds, B: phenylpropanoids, C: glycosides, D: phthalates, E: alkaloids, F: others.

### 3.2 JBD Suppresses the Activation of the TLR4 Signalling Pathway Triggered by LPS in Macrophages

**FIGURE 2:**
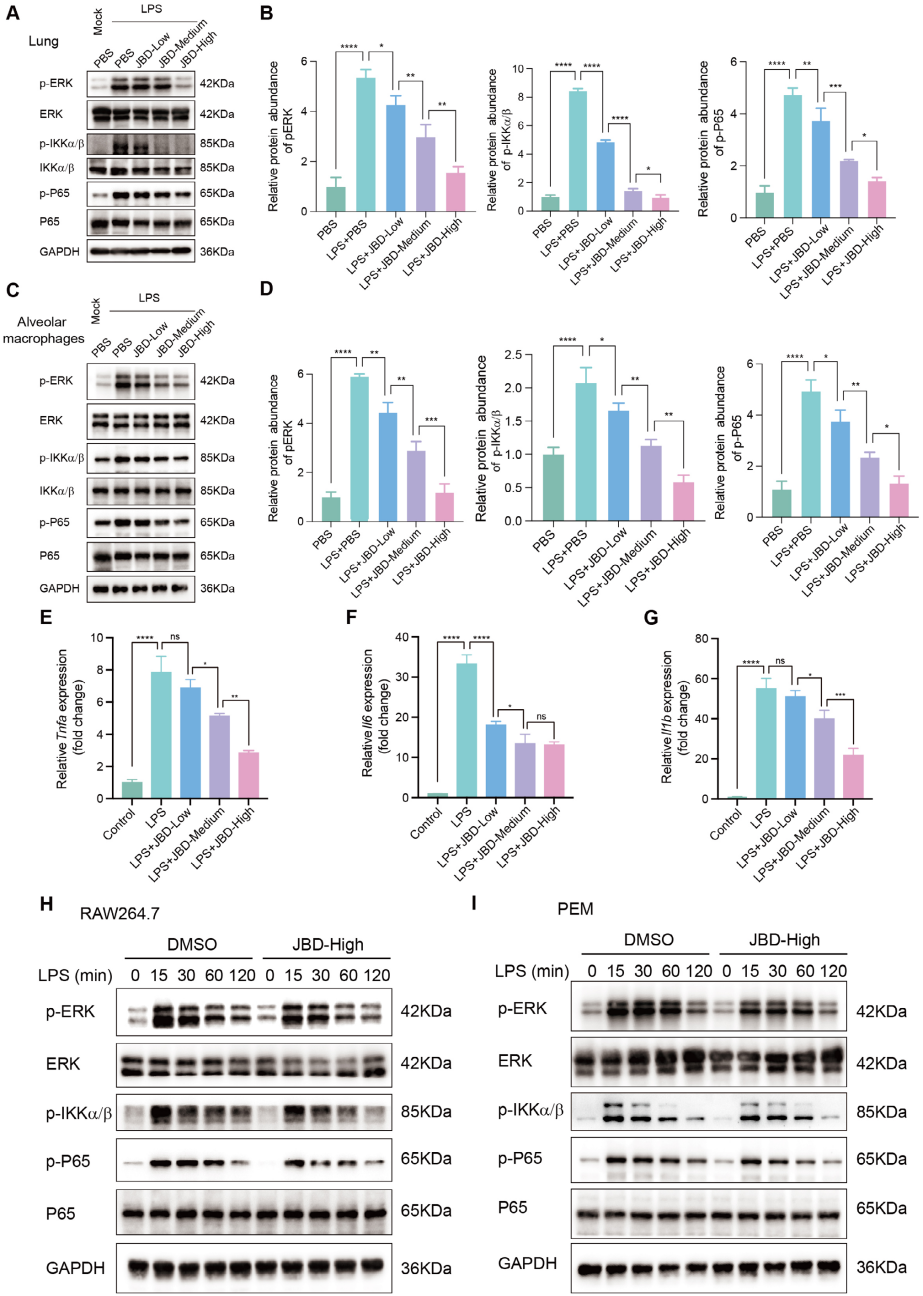
JBD modulates TLR4/NF‐κB signal transduction in alveolar macrophages. (A) Immunoblotting assessments of phosphorylated signalling mediators (p‐ERK, p‐IKKα/β and p‐p65) in pulmonary tissue lysates. (B) The quantitative analyses of p‐ERK, p‐IKKα/β and p‐p65 protein levels were normalised to their corresponding total protein expressions. (C) Parallel immunoblotting evaluations in purified alveolar macrophages demonstrated concentration‐dependent suppression of signalling cascade activation. (D) Phosphorylated ERK, IKKα/β and p65 levels were normalised to their respective total protein expressions in alveolar macrophages. (E–G). Molecular quantification of inflammatory mediator transcripts (Tnfα, Il6 and Il1β) in alveolar macrophages through quantitative RT‐PCR. Triplicate measurements were presented as mean ± SEM; *p < 0.05, **p < 0.01, ***p < 0.001, ****p < 0.0001 relative to LPS stimulation. (H, I). Experimental validation employing (H) RAW264.7 cell lines and (I) primary peritoneal macrophages exposed to JBD‐supplemented serum (10% v/v), revealing consistent blockade of LPS‐triggered (100 ng/mL) phosphorylation cascades. All immunoblot data reflect representative outcomes from three independent experimental trials.

Macrophages play an essential role in mediating LPS‐induced ALI through excessive inflammation. To validate the anti‐inflammatory mechanism of JBD via macrophage modulation, we subsequently investigated its effects on LPS‐induced inflammatory signalling pathways in macrophages. Given that TLR4 signalling serves as the primary pathway for LPS recognition and the activation of downstream inflammatory cascades, we first examined the activation status of this pathway in lung tissues 24 h after LPS challenge in our ALI mouse model. Our results demonstrated that JBD pretreatment significantly inhibited LPS‐induced activation of the TLR4 pathway in a dose‐dependent manner, as evidenced by reduced phosphorylation levels of ERK, IKKα/β and p65 (Figure 2A,B). We then isolated alveolar macrophages from the lung tissues of model mice to evaluate TLR4 signalling activation. Consistent with the findings in lung tissues, JBD treatment markedly suppressed LPS‐triggered activation of the TLR4 signalling pathway in alveolar macrophages, with higher doses showing the most pronounced inhibitory effect (Figure 2C,D). Moreover, in LPS‐stimulated macrophages, JBD dose‐dependently reduced the expression of TNF‐α, IL‐6 and IL‐1β, underscoring its macrophage‐specific anti‐inflammatory properties (Figure 2E–G).

We further utilised the RAW264.7 cell line and primary peritoneal macrophages (PEMs) as in vitro models to confirm the inhibitory effects of JBD on LPS‐induced inflammatory responses. First, JBD‐containing serum was prepared using SD rats. Briefly, the animals were intragastrically administered JBD (5 times concentrated) at low (0.63 mL/kg), medium (1.26 mL/kg), or high (2.52 mL/kg) doses once daily for seven consecutive days, whereas the control group was administered an equivalent volume of normal saline. Two hours after the final administration, the rats were humanely euthanized under anaesthesia. Whole blood was then collected, heat‐inactivated and sterile‐filtered to generate JBD‐containing serum for subsequent in vitro experiments. RAW264.7 and PEM cells were challenged by LPS 24 h post‐treatment by JBD‐containing serum. Consistent with the in vivo results, JBD‐containing serum markedly suppressed LPS‐triggered activation of the TLR4 signalling pathway, as demonstrated by decreased phosphorylation levels of key downstream signalling molecules of ERK, IKKα/β and p65 (Figure 2H,I). In conclusion, our data demonstrate that JBD alleviates LPS‐induced ALI by targeting macrophages to inhibit TLR4 activation, thereby reducing production of the related inflammatory cytokines.

### 3.3 JBD Attenuates the Release of Pro‐Inflammatory Cytokines by Macrophages in Response to LPS Stimulation

**FIGURE 3:**
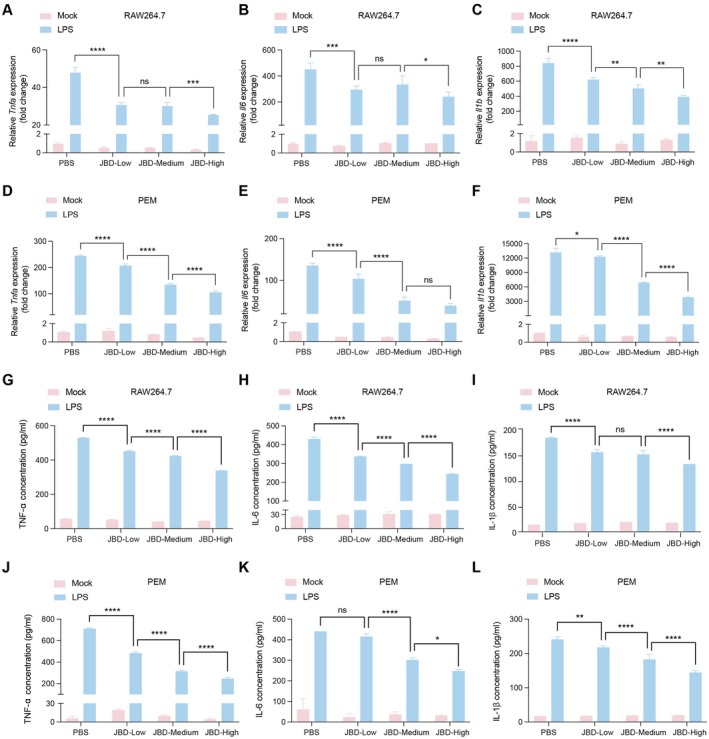
JBD inhibits LPS‐induced pro‐inflammatory cytokine secretion in macrophages. (A–C) Quantitative RT‐PCR profiling of cytokine transcripts, including *Tnfa* (A), *Il6* (B) and *Il1b* (C) in RAW264.7 cells pre‐treated by 10% JBD with different doses. (D–F) Quantitative RT‐PCR profiling of cytokine transcripts, including *Tnfa* (D), *Il6* (E) and *Il1b* (F) in PEM cells pre‐treated by 10% JBD with different doses. (G–I) ELISA quantification of released cytokines, including TNFα (G), IL6 (H) and IL‐1β (I) from RAW264.7 cell culture media. (J–L) ELISA quantification of released cytokines, including TNFα (J), IL6 (K) and IL‐1β (L) from PEM cell culture media. *p < 0.05, **p < 0.01, ***p < 0.001, and ****p < 0.0001 denote statistical significance relative to LPS stimulation.

To further confirm the inhibitory effects of JBD on LPS‐stimulated macrophages, we systematically assessed its regulatory influence on the production of inflammatory cytokines. RAW264.7 and PEM cells were pretreated for 24 h with 10% JBD‐containing serum at low, medium and high concentrations before being stimulated with LPS. At the transcriptional level, qRT‐PCR analysis revealed that JBD significantly reduced the expression of inflammatory cytokines induced by LPS in a dose‐dependent manner. In RAW264.7 cells, the high‐dose JBD treatment decreased the expression of TNF‐α, IL‐1β and IL‐6 by approximately 50%, 40% and 60%, respectively (Figure 3A–C). Similar inhibitory effects were observed in PEMs (Figure 3D–F), indicating the consistency of JBD's anti‐inflammatory activity across different macrophage models. Consistent with the transcriptional data, ELISA analysis of the culture supernatants demonstrated that JBD dramatically attenuated LPS‐stimulated release of TNF‐α, IL‐1β and IL‐6 in both RAW264.7 (Figure 3G–I) and PEM cells (Figure 3J–L). Taken together, these findings demonstrate that JBD effectively mitigates LPS‐triggered inflammatory responses, and most importantly, the magnitude of inhibition correlated with the dosage, with the most robust effects observed at the highest concentration of JBD‐containing serum.

### 3.4 Astrapterocarpan Is a Key Bioactive Component of JBD Responsible for Mediating Its Anti‐Inflammatory Effects

**FIGURE 4:**
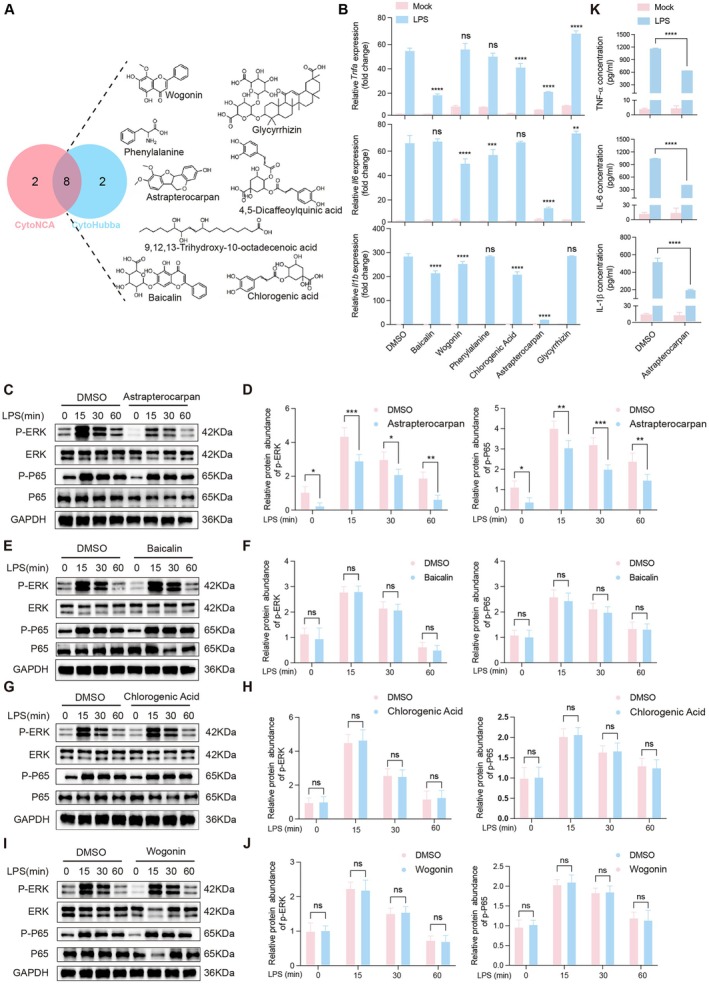
Screening of the bioactive constituent of JBD. (A) Shared candidate compounds identified through CytoNCA and CytoHubba computational approaches. (B) QRT‐PCR validation demonstrating astrapterocarpan's most potent suppression of cytokine mRNA levels versus other candidates (10 μM; ****p < 0.0001 vs. LPS). (C, E, G, I) Immunoblotting analyses of astrapterocarpan (C), baicalin (E), chlorogenic acid (G) and wogonin (I) effects on LPS induced activation of ERK and P65. (D, F, H, J) The quantitative analyses of p‐ERK, and p‐p65 protein levels were normalised to their corresponding total protein expressions affected by astrapterocarpan (D), baicalin (F), chlorogenic acid (H) and wogonin (J). (K) ELISA quantification of LPS‐induced TNFα, IL‐6 and IL‐1β levels in PEM cells following astrapterocarpan treatment. Data were presented as mean ± SEM, *n* = 3; with *p < 0.05, **p < 0.01, ***p < 0.001, ****p < 0.0001.

Our study revealed that JBD mitigates LPS‐induced ALI by suppressing the TLR4/NF‐κB signalling pathway and decreasing the production of inflammatory cytokines in macrophages. To identify the principal bioactive constituents underlying these effects, we implemented an integrated analytical methodology incorporating CytoNCA and CytoHubba algorithms with *Z*‐score prioritisation technology. Both analytical approaches independently identified ten core components, with an overlap of eight shared compounds (Figure 4A). Among these, six commercially available compounds, baicalin, wogonin, phenylalanine, chlorogenic acid, astrapterocarpan and glycyrrhizin, were selected for further functional validation.

To evaluate their anti‐inflammatory potential, PEMs were pretreated with each of the six candidate compounds prior to LPS stimulation. qRT‐PCR analysis revealed that phenylalanine and glycyrrhizin had no inhibitory effect on the expression of the assessed cytokines. Baicalin and chlorogenic acid showed mild suppression of TNF‐α and IL‐1β expression, while wogonin marginally reduced IL‐6 and IL‐1β levels. Notably, astrapterocarpan demonstrated the most significant inhibitory effects on all three cytokines examined, suggesting its specific modulation of the inflammatory signalling pathway and a distinct mechanism underlying its anti‐inflammatory activity (Figure 4B). We further investigated the modulation of TLR4 signalling by these four active compounds. Western blot analysis indicated that astrapterocarpan uniquely attenuated LPS‐induced phosphorylation of both ERK and p65 (Figure 4C,D), whereas the other three compounds showed minimal effects (Figure 4E–J). This targeted suppression of TLR4 downstream signalling effectors highlights the distinctive anti‐inflammatory contribution of astrapterocarpan to the pharmacological effects of JBD. Consistently, astrapterocarpan administration also markedly suppressed the LPS‐stimulated release of TNF‐α, IL‐6 and IL‐1β in PEMs (Figure 4K), further supporting its specific inhibitory activity on the TLR4/NF‐κB signalling axis.

### 3.5 Screening the Binding Targets of Astrapterocarpan in LPS‐TLR4 Signalling Pathway

**FIGURE 5:**
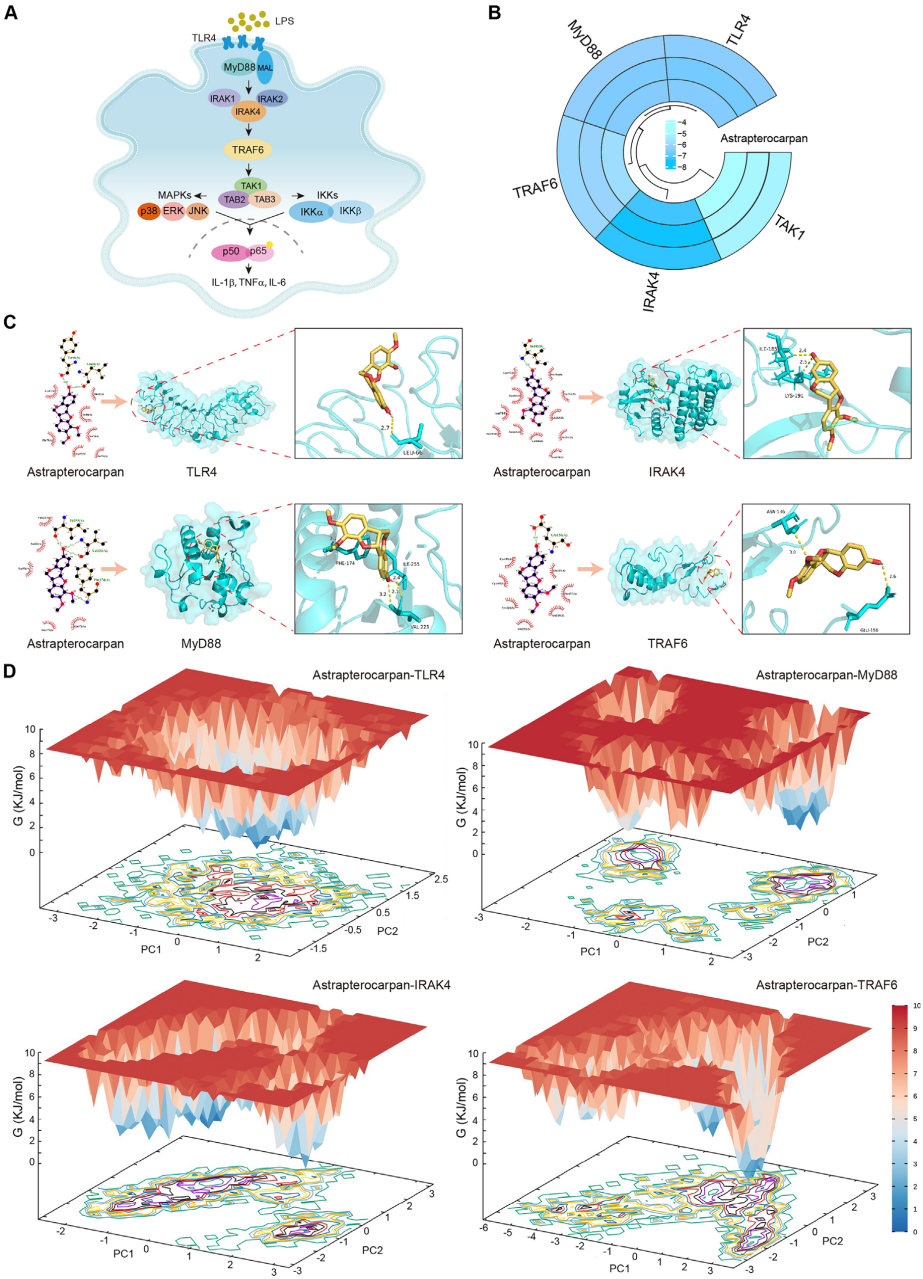
The prediction of potential binding targets of astrapterocarpan. (A) Schematic of TLR4‐MyD88‐TRAF6‐TAK1‐NF‐κB/MAPK pathway. (B) Heatmap of molecular docking binding energies (kcal/mol), demonstrating astrapterocarpan's binding affinity for the indicated proteins, including TLR4, MyD88, IRAK4, TRAF6 and TAK1. (C) Computational modelling (PyMOL) illustrating astrapterocarpan's interaction patterns with TLR4, MyD88, IRAK4 and TRAF6 residues. (D) Free energy landscape (FEL) analysis demonstrated the conformational state of the four proteins (TLR4, MyD88, IRAK4 and TRAF6) upon the compound binding, as indicated by changes in global energy distribution.

The activation of TLR4 signalling is mediated by a cascade of essential adaptor proteins. To identify the molecular targets of astrapterocarpan within the LPS‐TLR4 signalling axis, we focused on five central pathway components: TLR4, MyD88, IRAK4, TRAF6 and TAK1 (Figure 5A). To identify the potential targets of astrapterocarpan, we initially conducted molecular docking simulations in triplicate for each protein–astrapterocarpan pair. The binding affinities were determined by calculating the mean binding energies and corresponding standard deviations from three independent runs, thereby ensuring the reliability and reproducibility of the results. These data were summarised in a heatmap, where a pink‐to‐blue colour gradient illustrated the range of binding affinities across targets (Figure 5B). Applying a binding energy threshold of −5.0 kcal/mol as indicative of biologically relevant interactions, our analysis revealed that astrapterocarpan formed highly stable complexes (binding energies < −6.0 kcal/mol) with all four target proteins in the molecular docking simulations, except for TAK1 (Figure 5C). The Free Energy Landscape (FEL) analysis demonstrates that astrapterocarpan can significantly modulate the global energy states of TLR4, MyD88, IRAK4 and TRAF6, promoting a transition toward a more stable conformational state, which suggests a high likelihood of interaction between astrapterocarpan and these four proteins (Figure 5D).

### 3.6 Astrapterocarpan Directly Targets TRAF6 in the TLR4 Signalling Pathway

**FIGURE 6:**
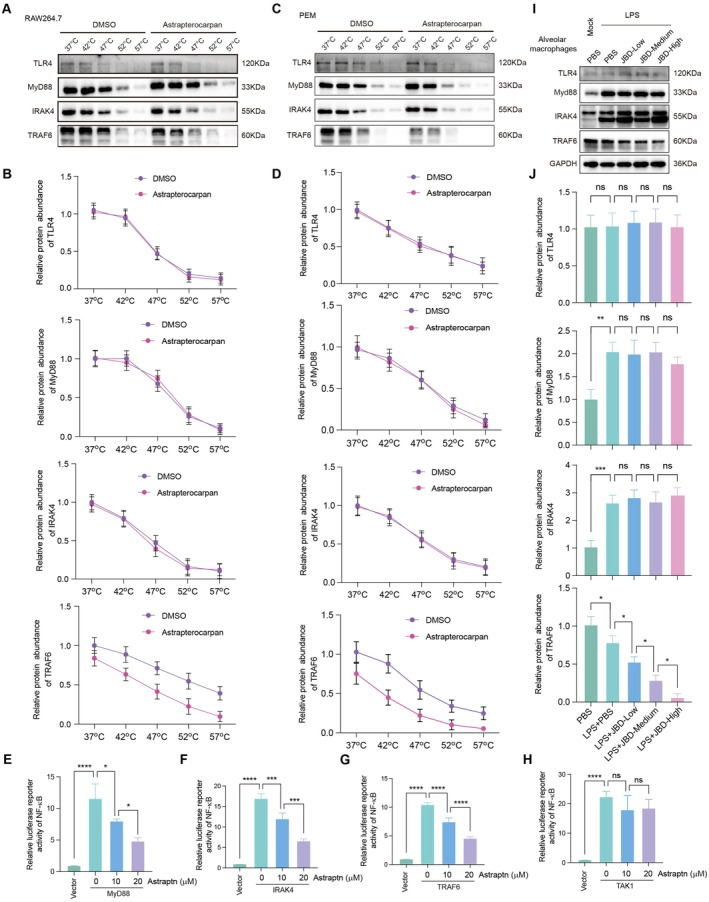
Astrapterocarpan specifically interacts with TRAF6. (A, C) Cellular thermal shift assays (CETSA) of TRAF6 in (A) RAW264.7 macrophages and (C) PEMs treated with or without Astrapterocarpan (20 μM). Lysates were subjected to temperature gradient followed by western blot analysis (*p < 0.05, **p < 0.01 vs. untreated controls). (B, D) The quantitative analyses of TLR4, MyD88, IRAK4 and TRAF6 protein levels were performed by calculating the grayscale values, which were normalised to the values obtained under the condition of 37°C for both RAW264.7 (B) and PEM cells (D). (E–H) Luciferase reporter plasmids containing the NF‐κB promoter were co‐transfected with expression plasmids encoding the indicated proteins, including MyD88 (E), IRAK4 (F), TRAF6 (G) and TAK1 (H), into RAW264.7 cells. After a 24‐h incubation period post‐transfection, the cells were collected and luciferase activity was assessed using a luciferase reporter assay kit. (I) Alveolar macrophages were isolated from LPS‐induced mice that had been administered different doses of JBD via intragastric gavage to evaluate the expression levels of the indicated proteins, including TLR4, MyD88, IRAK4 and TRAF6. (J) Quantitative analyses of TLR4, MyD88, IRAK4 and TRAF6 protein levels were normalised relative to GAPDH expression. Astraptn: Astrapterocarpan. *p < 0.05, ***p < 0.001, ****p < 0.0001.

With the aim to further decipher whether the potential interaction could influence the stability of the target proteins, we first conducted the cellular thermal shift assay (CETSA). Western blot analysis demonstrated that astrapterocarpan treatment significantly decreased the protein stability of TRAF6 in both RAW264.7 and PEM cells, while no significant effect was observed on the stability of TLR4, MyD88 or IRAK4 (Figure 6A,C). The quantitative analysis of protein band intensities further indicates that, under astrapterocarpan treatment conditions, TRAF6 exhibits reduced thermal tolerance, whereas the thermal stability of the other three proteins remains largely unaffected (Figure 6B,D). These findings suggest that astrapterocarpan may specifically target TRAF6 to exert its anti‐inflammatory effects. We subsequently performed a luciferase reporter assay to validate that astrapterocarpan specifically targets TRAF6. The indicated plasmids were transfected into RAW264.7 cells to induce an increase in NF‐κB promoter activity mediated by MyD88, IRAK4, TRAF6 and TAK1, and the subsequent attenuation of NF‐κB activity modulated by astrapterocarpan was then assessed. The results demonstrated that astrapterocarpan could dose‐dependently reduce the NF‐κB promoter activity induced by MyD88, IRAK4 and TRAF6, but had no effect on TAK1‐induced NF‐κB activation (Figure 6E–H). Given that TRAF6 serves as a central adaptor protein downstream of MyD88 and IRAK4 and upstream of TAK1, these findings confirm that astrapterocarpan targets TRAF6.

To further confirm that astrapterocarpan‐mediated TRAF6 inhibition constitutes a key mechanism underlying JBD's protective effects against LPS‐induced ALI, we analysed the expression levels of key TLR4 pathway components (TRAF6, TLR4, MyD88 and IRAK4) in alveolar macrophages isolated from the murine model. Consistent with the CETSA findings, JBD treatment significantly reduced TRAF6 protein levels in LPS‐challenged mice, with minimal impact on the expression of TLR4, MyD88 or IRAK4 (Figure 6I,J). Collectively, these findings from both computational and experimental approaches demonstrate that astrapterocarpan serves as the core bioactive constituent of JBD by selectively binding to and destabilising TRAF6, thereby potentially interfering with its role in NF‐κB activation downstream of TLR4 signalling in macrophages.

### 3.7 Astrapterocarpan Attenuates LPS‐Induced Acute Lung Injury In Vivo

**FIGURE 7:**
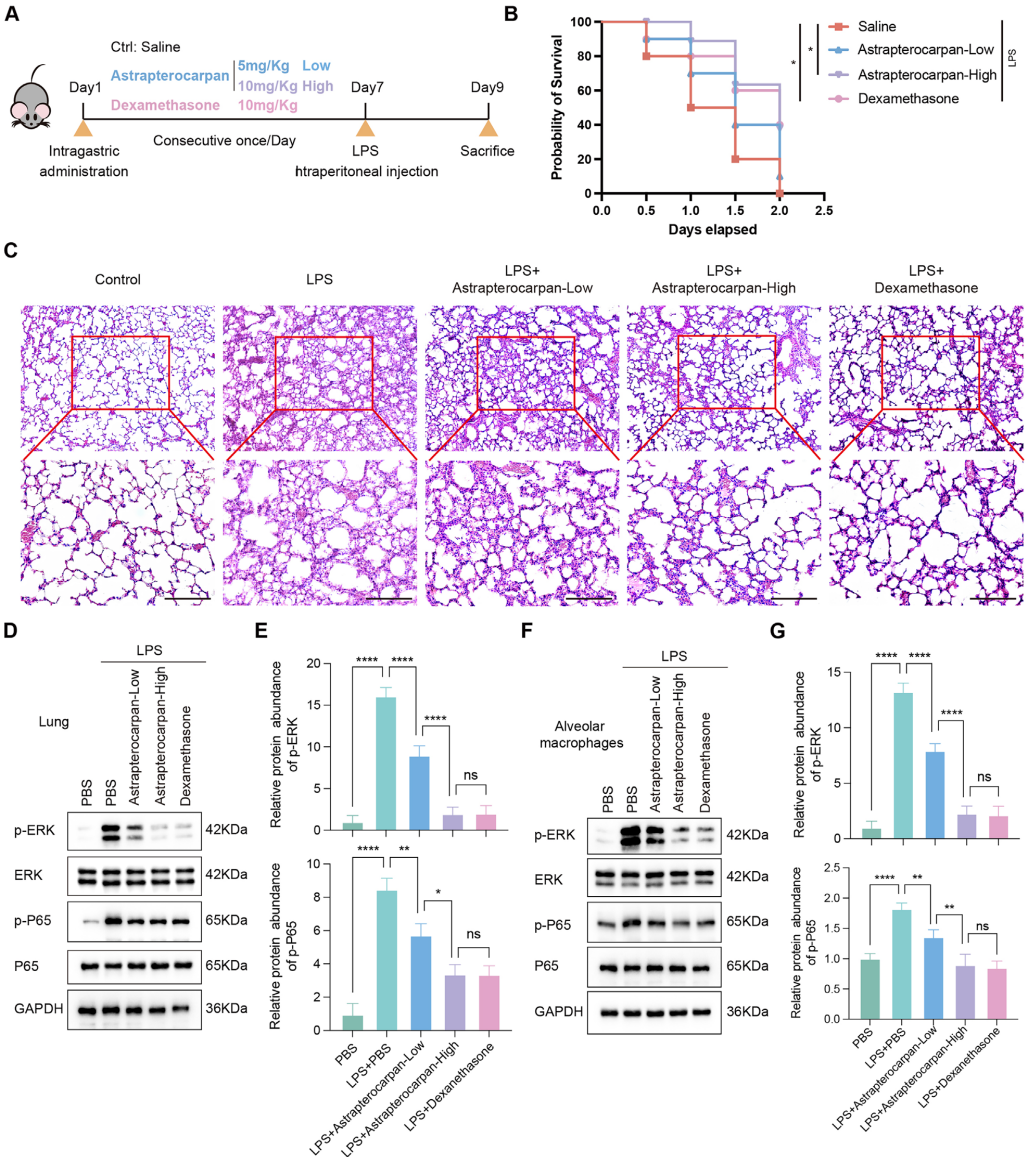
Astrapterocarpan attenuates LPS‐induced ALI in vivo. (A) The experimental design is illustrated as follows: Mice received pretreatment with astrapterocarpan (5/10 mg/kg) or dexamethasone (10 mg/kg) prior to LPS challenge. (B) Survival analysis showed high‐dose astrapterocarpan achieved comparable efficacy to dexamethasone in extending survival duration (48‐h observation period; *n* = 10 animals per group). (C) Histopathological evaluation through H&E staining (scale bars = 100 μm) revealed a dose‐responsive reduction in pulmonary injury severity. (D, E) The lung tissues were collected from LPS‐induced mice that had been administrated different doses of astrapterocarpan to evaluate the activation of ERK and p65 by western blot (D), and the quantitative analyses of p‐ERK, and p‐p65 protein levels were normalised to their corresponding total protein expressions affected by astrapterocarpan (E). (F, G) Alveolar macrophages were isolated from LPS‐induced mice that had been administered different doses of astrapterocarpan to evaluate the activation of ERK and p65 by western blot (F), and the quantitative analyses of p‐ERK, and p‐p65 protein levels were normalised to their corresponding total protein expressions affected by astrapterocarpan (G). All experimental results present mean ± SEM values from ≥ 3 independent trials, with *p < 0.05, **p < 0.01, ****p < 0.0001.

To assess the in vivo therapeutic potential of astrapterocarpan, we employed the LPS‐induced mice model of ALI with three treatment groups: low‐dose astrapterocarpan (5 mg/kg), high‐dose astrapterocarpan (10 mg/kg) and dexamethasone (10 mg/kg) as a positive control (Figure 7A). Both astrapterocarpan treatment groups showed significantly improved survival rates compared to the LPS‐only controls, demonstrating protective effects comparable to those of dexamethasone (Figure 7B). Histopathological analysis revealed that astrapterocarpan dose‐dependently alleviated key features of lung injury, including thickened alveolar walls, infiltration of inflammatory cells and haemorrhage (Figure 7C). Notably, the high‐dose astrapterocarpan group exhibited protective functions that were comparable to those observed in the dexamethasone‐treated group.

To gain mechanistic insights into its protective effects, we further investigated astrapterocarpan's modulation of LPS‐mediated TLR4 signalling. Western blot analysis showed dose‐dependent suppression of LPS‐induced ERK and p65 phosphorylation in both lung tissues and alveolar macrophages, indicating concurrent inhibition of the MAPK and NF‐κB signalling pathways (Figure 7D,F). Protein quantification analysis further confirmed that astrapterocarpan exerts regulatory effects on the TLR4 signalling pathway (Figure 7E,G). These results clearly indicate that astrapterocarpan attenuates ALI through selective inhibition of TRAF6‐dependent signalling, with the high‐dose regimen showing efficacy comparable to that of dexamethasone. Importantly, our findings provide compelling evidence that the therapeutic effects of astrapterocarpan are attributable to its suppression of the TRAF6‐mediated inflammatory signalling pathway.

## 4 Discussion

The present findings demonstrate that JBD exerts substantial protective effects against LPS‐induced ALI by modulating macrophage‐mediated inflammatory responses. Administration of JBD prior to the LPS challenge enhances survival rates, alleviates histopathological alterations in lung tissue, and lowers systemic levels of pro‐inflammatory cytokines, such as TNF‐α, IL‐6 and IL‐1β, in a dose‐dependent manner. Mechanistically, JBD attenuates inflammation through the inhibition of the TLR4/NF‐κB signalling pathway in alveolar macrophages, leading to the suppression of the excessive inflammatory response that characterises ALI pathophysiology. Notably, astrapterocarpan was identified as the principal bioactive constituent of JBD, which selectively interacts with TRAF6 to interfere with downstream inflammatory signalling cascades. These results provide novel mechanistic understanding of the therapeutic potential of JBD and its active components in the treatment of ALI, highlighting the integration of traditional herbal medicine with contemporary pharmacological strategies.

A key discovery of this study is that JBD exerts specific regulation on macrophages, thereby influencing the pathogenesis of ALI. Macrophages are critically involved in the development and progression of ALI, as their polarisation toward a pro‐inflammatory phenotype contributes to the cytokine storm and subsequent tissue damage via activation of the TLR4/NF‐κB and MAPK signalling pathways, resulting in the excessive production of TNF‐α, IL‐6 and IL‐1β [32, 33]. These inflammatory cytokines compromise endothelial barrier integrity, promote neutrophil recruitment and exacerbate pulmonary edema and oxidative stress [34]. Our data show that JBD effectively inhibits LPS‐induced macrophage activation by targeting these signalling cascades, as demonstrated by reduced phosphorylation of ERK, IKKα/β and p65, key mediators of inflammatory signal transduction, in both alveolar macrophages isolated from in vivo models and cultured macrophage cell lines. Importantly, this inhibitory effect is closely associated with decreased cytokine production and may further reduce neutrophil infiltration in lung tissues, thereby interrupting the pathological macrophage–neutrophil crosstalk that drives ALI progression. This macrophage‐targeted mechanism may account for JBD's capacity to alleviate lung injury without impairing essential immune defences, thereby addressing a major limitation of existing anti‐inflammatory therapies.

The identification of astrapterocarpan as the primary bioactive constituent of JBD marks a significant advance in elucidating its molecular mechanism of action. Through an integrated approach combining network pharmacology and experimental validation, we demonstrated that astrapterocarpan selectively binds to TRAF6, a central adaptor protein in the TLR4 signalling pathway. This interaction results in the destabilisation of TRAF6 and blocks its subsequent activation of the NF‐κB and MAPK signalling pathways, effectively interrupting the inflammatory cascade at a pivotal regulatory node [35]. The specificity of this interaction is particularly remarkable, as astrapterocarpan exhibited minimal effects on upstream components such as TLR4, MyD88 or IRAK4, suggesting a targeted mechanism that may minimise off‐target effects. Moreover, in vivo studies confirmed that astrapterocarpan alone can recapitulate the protective effects of JBD, demonstrating efficacy comparable to that of dexamethasone in improving survival rates and attenuating lung injury. Notably, JBD was also found to reduce TRAF6 expression in alveolar macrophages, which likely disrupts the self‐sustaining loop of macrophage activation. TRAF6 not only mediates the activation of NF‐κB and MAPKs but also contributes to inflammasome priming through ASC ubiquitination [36]. By destabilising TRAF6, astrapterocarpan may broadly suppress multiple inflammatory checkpoints driven by macrophages, offering a distinct advantage over inhibitors that target only a single signalling pathway. These findings not only confirm astrapterocarpan as the key therapeutic component of JBD but also underscore its potential as a promising standalone therapeutic agent.

From a translational point, our findings address key unmet needs in the management of ALI by demonstrating the therapeutic potential of JBD as a targeted intervention. Current treatment strategies remain predominantly supportive, with a lack of effective modalities that directly modulate the underlying inflammatory pathology [37]. The multi‐component composition of JBD offers a distinct advantage by concurrently targeting TLR4/NF‐κB‐mediated injury pathways, potentially enhancing therapeutic efficacy, by multi‐level regulation. Importantly, we identified astrapterocarpan as a core bioactive constituent of JBD, which, as a single compound, exhibits dose‐dependent protective effects comparable to those of dexamethasone in reducing mortality and pulmonary damage. This discovery not only substantiates the mechanistic basis of JBD's action but also lays the groundwork for the development of novel, well‐defined monotherapies with improved pharmacokinetic profiles. Future studies should focus on optimising delivery systems, exploring potential synergies with existing therapeutic regimens, and evaluating efficacy in related TLR4‐driven conditions such as sepsis‐induced acute respiratory distress syndrome (ARDS). These advancements hold promise for improving clinical outcomes in ALI patients and extending therapeutic applications to other inflammatory lung disorders.

Our study reveals a novel mechanism through which JBD and its bioactive constituent astrapterocarpan confer protection against ALI by selectively inhibiting TRAF6‐dependent macrophage activation. These findings not only enhance our understanding of ALI pathophysiology but also illustrate how traditional herbal medicine can inform the development of precise, molecularly targeted therapeutic strategies. By integrating traditional medicine with contemporary pharmacological approaches, this work establishes a conceptual framework for the discovery and development of novel immunomodulatory agents that effectively balance therapeutic efficacy and safety in severe inflammatory conditions. Future research should prioritise clinical translation and further investigate the potential applicability of these insights to a broader range of inflammatory diseases.

## 5 Conclusions

In conclusion, this study demonstrates that JBD and its principal bioactive constituent, astrapterocarpan, ameliorate LPS‐induced ALI through targeted immunomodulation of macrophage‐mediated inflammatory responses. JBD exerts its protective effects by selectively inhibiting the TLR4/TRAF6/NF‐κB/MAPK signalling cascade in macrophages, thereby suppressing excessive cytokine production, reducing alveolar infiltration of inflammatory cells, preserving pulmonary architecture and enhancing survival. Astrapterocarpan, identified as the key therapeutic component, mediates its anti‐inflammatory activity through direct interaction with TRAF6, providing a mechanism‐based alternative to conventional broad‐spectrum corticosteroids. Notably, JBD's multi‐component formulation integrates the holistic principles of traditional medicine with precise molecular targeting, effectively interrupting pathological inflammatory processes. These findings highlight the therapeutic potential of macrophage‐targeted interventions in the management of ALI. Further clinical investigations are necessary to translate these preclinical observations into the heterogeneous clinical context of sepsis‐associated ALI.

## Author Contributions

Xia Li and Zhen Zhang: investigation, design, writing and editing, funding acquisition. Wei Li, Aijun Zhang, Yongqing Cai and Haoyu Sun: method design, systematic analysis and composing the original draft. Yao Teng: investigation, validation, visualisation. Zhaoqing Meng and Weiwei Zhou: investigation, formal analysis. Ruixin Liu: methodology, investigation. Jingzhen Tian: conceptualization and article polishing.

## Ethics Statement

All experimental protocols involving mice and rats were carried out in accordance with the standards set by the Association for Assessment and Accreditation of Laboratory Animal Care and were approved by the Animal Ethics Committee of Shandong University of Traditional Chinese Medicine (Jinan, Shandong Province, China) (Permit number: SDUTCM20240604001 for mouse, SDUTCM20240812001 for rat).

## Conflicts of Interest

The authors declare no conflicts of interest.

## Data Availability

The data that support the findings of this study are available from the corresponding author upon reasonable request.

## References

[1] Z. Ren , Z. Zheng , and X. Feng , “Role of Gut Microbes in Acute Lung Injury/Acute Respiratory Distress Syndrome,” Gut Microbes 16 (2024): 2440125. 39658851 10.1080/19490976.2024.2440125 PMC11639474

[2] B. Li , J. Liu , W. He , et al., “Inhibition of Macrophage Inflammasome Assembly and Pyroptosis With GC‐1 Ameliorates Acute Lung Injury,” Theranostics 15 (2025): 2360–2374. 39990234 10.7150/thno.101866 PMC11840730

[3] K. E. Rudd , S. C. Johnson , K. M. Agesa , et al., “Global, Regional, and National Sepsis Incidence and Mortality, 1990–2017: Analysis for the Global Burden of Disease Study,” Lancet 395 (2020): 200–211. 31954465 10.1016/S0140-6736(19)32989-7 PMC6970225

[4] X. Zhou and Y. Liao , “Gut‐Lung Crosstalk in Sepsis‐Induced Acute Lung Injury,” Frontiers in Microbiology 12 (2021): 779620. 35003009 10.3389/fmicb.2021.779620 PMC8733643

[5] Y. Jiang , S. Gao , Z. Chen , et al., “Pyroptosis in Septic Lung Injury: Interactions With Other Types of Cell Death,” Biomedicine & Pharmacotherapy 169 (2023): 115914. 38000360 10.1016/j.biopha.2023.115914

[6] J. Reutershan , M. A. Morris , T. L. Burcin , et al., “Critical Role of Endothelial CXCR2 in LPS‐Induced Neutrophil Migration Into the Lung,” Journal of Clinical Investigation 116 (2006): 695–702. 16485040 10.1172/JCI27009 PMC1366502

[7] Y. Wang , X. Wang , Y. Li , et al., “Xuanfei Baidu Decoction Reduces Acute Lung Injury by Regulating Infiltration of Neutrophils and Macrophages via PD‐1/IL17A Pathway,” Pharmacological Research 176 (2022): 106083. 35033647 10.1016/j.phrs.2022.106083 PMC8757644

[8] H. Hong , Y. Wu , Y. Li , et al., “Endothelial PPARdelta Ablation Exacerbates Vascular Hyperpermeability via STAT1/CXCL10 Signaling in Acute Lung Injury,” Circulation Research 136 (2025): 735–751. 39996324 10.1161/CIRCRESAHA.124.325855

[9] S. Y. Chen , Y. L. Chen , P. C. Li , et al., “Engineered Extracellular Vesicles Carrying Let‐7a‐5p for Alleviating Inflammation in Acute Lung Injury,” Journal of Biomedical Science 31 (2024): 30. 38500170 10.1186/s12929-024-01019-4 PMC10949767

[10] B. Li , C. Xia , W. He , et al., “The Thyroid Hormone Analog GC‐1 Mitigates Acute Lung Injury by Inhibiting M1 Macrophage Polarization,” Advanced Science 11 (2024): e2401931. 39373388 10.1002/advs.202401931 PMC11600256

[11] Y. Okabe and R. Medzhitov , “Tissue Biology Perspective on Macrophages,” Nature Immunology 17 (2016): 9–17. 26681457 10.1038/ni.3320

[12] M. Guilliams and F. R. Svedberg , “Does Tissue Imprinting Restrict Macrophage Plasticity?,” Nature Immunology 22 (2021): 118–127. 33462453 10.1038/s41590-020-00849-2

[13] H. Zhong , J. Ji , J. Zhuang , et al., “Tissue‐Resident Macrophages Exacerbate Lung Injury After Remote Sterile Damage,” Cellular & Molecular Immunology 21 (2024): 332–348. 38228746 10.1038/s41423-024-01125-1 PMC10979030

[14] Z. Zhang , L. Zhang , B. Wang , et al., “RNF144B Inhibits LPS‐Induced Inflammatory Responses via Binding TBK1,” Journal of Leukocyte Biology 106 (2019): 1303–1311. 31509299 10.1002/JLB.2A0819-055R PMC6899866

[15] C. Malainou , S. M. Abdin , N. Lachmann , U. Matt , and S. Herold , “Alveolar Macrophages in Tissue Homeostasis, Inflammation, and Infection: Evolving Concepts of Therapeutic Targeting,” Journal of Clinical Investigation 133 (2023): e170501. 37781922 10.1172/JCI170501 PMC10541196

[16] Y. J. Fu , B. Xu , S. W. Huang , et al., “Baicalin Prevents LPS‐Induced Activation of TLR4/NF‐kappaB p65 Pathway and Inflammation in Mice via Inhibiting the Expression of CD14,” Acta Pharmacologica Sinica 42 (2021): 88–96. 32457419 10.1038/s41401-020-0411-9 PMC7921675

[17] A. Ciesielska , M. Matyjek , and K. Kwiatkowska , “TLR4 and CD14 Trafficking and Its Influence on LPS‐Induced Pro‐Inflammatory Signaling,” Cellular and Molecular Life Sciences 78 (2021): 1233–1261. 33057840 10.1007/s00018-020-03656-y PMC7904555

[18] Y. C. Wu , S. P. Hsu , M. C. Hu , Y. T. Lan , E. T. H. Yeh , and F. M. Yang , “PEP‐sNASP Peptide Alleviates LPS‐Induced Acute Lung Injury Through the TLR4/TRAF6 Axis,” Frontiers in Medicine 9 (2022): 832713. 35386914 10.3389/fmed.2022.832713 PMC8977741

[19] M. Pereira , D. F. Durso , C. E. Bryant , et al., “The IRAK4 Scaffold Integrates TLR4‐Driven TRIF and MYD88 Signaling Pathways,” Cell Reports 40 (2022): 111225. 35977521 10.1016/j.celrep.2022.111225 PMC9446533

[20] M. Hinz and C. Scheidereit , “The IkappaB Kinase Complex in NF‐kappaB Regulation and Beyond,” EMBO Reports 15 (2014): 46–61. 24375677 10.1002/embr.201337983 PMC4303448

[21] A. Oeckinghaus , M. S. Hayden , and S. Ghosh , “Crosstalk in NF‐kappaB Signaling Pathways,” Nature Immunology 12 (2011): 695–708. 21772278 10.1038/ni.2065

[22] D. S. Cheng , W. Han , S. M. Chen , et al., “Airway Epithelium Controls Lung Inflammation and Injury Through the NF‐Kappa B Pathway,” Journal of Immunology (Baltimore, Md.: 1950) 178 (2007): 6504–6513. 17475880 10.4049/jimmunol.178.10.6504

[23] L. Li , W. Wu , W. Huang , G. Hu , W. Yuan , and W. Li , “NF‐kappaB RNAi Decreases the Bax/Bcl‐2 Ratio and Inhibits TNF‐Alpha‐Induced Apoptosis in Human Alveolar Epithelial Cells,” Inflammation Research 62 (2013): 387–397. 23334076 10.1007/s00011-013-0590-7

[24] D. Abrams , C. Agerstrand , J. R. Beitler , et al., “Risks and Benefits of Ultra‐Lung‐Protective Invasive Mechanical Ventilation Strategies With a Focus on Extracorporeal Support,” American Journal of Respiratory and Critical Care Medicine 205 (2022): 873–882. 35044901 10.1164/rccm.202110-2252CP

[25] F. D. Simonis , S. Einav , A. Serpa Neto , et al., “Epidemiology, Ventilation Management and Outcome in Patients Receiving Intensive Care After Non‐Thoracic Surgery—Insights From the LAS VEGAS Study,” Pulmonology 28 (2022): 90–98. 34906445 10.1016/j.pulmoe.2021.10.004

[26] C. Guerin , L. Baboi , and J. C. Richard , “Mechanisms of the Effects of Prone Positioning in Acute Respiratory Distress Syndrome,” Intensive Care Medicine 40 (2014): 1634–1642. 25266133 10.1007/s00134-014-3500-8

[27] Z. Zhai , W. Ouyang , Y. Yao , et al., “Dexamethasone‐Loaded ROS‐Responsive Poly(Thioketal) Nanoparticles Suppress Inflammation and Oxidative Stress of Acute Lung Injury,” Bioactive Materials 14 (2022): 430–442. 35415281 10.1016/j.bioactmat.2022.01.047 PMC8965854

[28] G. U. Meduri , D. Annane , M. Confalonieri , et al., “Pharmacological Principles Guiding Prolonged Glucocorticoid Treatment in ARDS,” Intensive Care Medicine 46 (2020): 2284–2296. 33150472 10.1007/s00134-020-06289-8 PMC7641258

[29] X. Y. Xing , W. J. Qiang , J. L. Bao , et al., “Jinbei Oral Liquid Ameliorates Bleomycin‐Induced Idiopathic Pulmonary Fibrosis in Rats via Reversion of Th1/Th2 Shift,” Chinese Herbal Medicines 12 (2020): 273–280. 36119009 10.1016/j.chmed.2020.05.002 PMC9476682

[30] A. Zhang , Y. Zou , Q. Xu , et al., “Investigation of the Pharmacological Effect and Mechanism of Jinbei Oral Liquid in the Treatment of Idiopathic Pulmonary Fibrosis Using Network Pharmacology and Experimental Validation,” Frontiers in Pharmacology 13 (2022): 919388. 35784749 10.3389/fphar.2022.919388 PMC9240387

[31] A. Zhang , K. Han , F. Chen , et al., “Jinbei Oral Liquid for Idiopathic Pulmonary Fibrosis: A Randomized Placebo‐Controlled Trial,” Scientific Reports 15 (2025): 3007. 39849152 10.1038/s41598-025-87474-x PMC11759330

[32] M. Shan , S. Zhang , Z. Luo , et al., “Itaconate Promotes Inflammatory Responses in Tissue‐Resident Alveolar Macrophages and Exacerbates Acute Lung Injury,” Cell Metabolism 37 (2025): 1750–1765.e1757. 40527316 10.1016/j.cmet.2025.05.012

[33] H. S. An , J. W. Yoo , J. H. Jeong , et al., “Lipocalin‐2 Promotes Acute Lung Inflammation and Oxidative Stress by Enhancing Macrophage Iron Accumulation,” International Journal of Biological Sciences 19 (2023): 1163–1177. 36923935 10.7150/ijbs.79915 PMC10008694

[34] H. Hong , S. Lou , F. Zheng , et al., “Hydnocarpin D Attenuates Lipopolysaccharide‐Induced Acute Lung Injury via MAPK/NF‐kappaB and Keap1/Nrf2/HO‐1 Pathway,” Phytomedicine 101 (2022): 154143. 35537248 10.1016/j.phymed.2022.154143

[35] A. Dawuti , S. Sun , R. Wang , et al., “Salvianolic Acid A Alleviates Heart Failure With Preserved Ejection Fraction via Regulating TLR/Myd88/TRAF/NF‐kappaB and p38MAPK/CREB Signaling Pathways,” Biomedicine & Pharmacotherapy 168 (2023): 115837. 37931518 10.1016/j.biopha.2023.115837

[36] G. Lopez‐Castejon , “Control of the Inflammasome by the Ubiquitin System,” FEBS Journal 287 (2020): 11–26. 31679183 10.1111/febs.15118 PMC7138099

[37] D. Mokra and J. Mokry , “Phosphodiesterase Inhibitors in Acute Lung Injury: What Are the Perspectives?,” International Journal of Molecular Sciences 22 (2021): 1929. 33669167 10.3390/ijms22041929 PMC7919656

